# Connexins 43 and 45 hemichannels mediate ATP release in the urinary bladder

**DOI:** 10.14440/bladder.0125

**Published:** 2025-08-21

**Authors:** Hafiz Sana-Ur-Rehman, Irit Markus, Gila Moalem-Taylor, Kate H. Moore, Kylie J. Mansfield, Lu Liu

**Affiliations:** 1 Department of Pharmacology, School of Biomedical Sciences, University of New South Wales, Sydney, New South Wales, NSW 2052, Australia; 2 Translational Neuroscience Facility, School of Biomedical Sciences, University of New South Wales, Sydney, New South Wales, NSW 2052, Australia; 3 Department of Urogynaecology, St George Hospital, University of New South Wales, Sydney, New South Wales, NSW 2217, Australia; 4 Graduate School of Medicine, University of Wollongong, New South Wales, NSW 2522, Australia

**Keywords:** Bladder, Urothelium, ATP release, Connexins, Stretch, Calcium ion depletion

## Abstract

**Background:**

Connexin (Cx) proteins form gap junctions between adjacent cells to facilitate intercellular communication and also assemble into hemichannels that release small molecules, including adenosine triphosphate (ATP), into the extracellular microenvironment, where ATP acts on purinergic receptors.

**Objective:**

This study investigated the roles of Cx43 and Cx45 as ATP release channels in the urinary bladder.

**Methods:**

Porcine bladder tissues and cultured cells were stained for Cx43 and Cx45 using immunofluorescence. Cx43- and Cx45-mediated ATP release in response to hypotonic stretch and extracellular Ca^2+^ depletion was assessed in porcine urothelial, suburothelial, and detrusor muscle cells, as well as in the human RT4 cell line.

**Results:**

The expression of Cx43 and Cx45 was Immunohistochemically confirmed in porcine bladder tissue, cultured porcine bladder urothelial cells, suburothelial myofibroblasts, detrusor muscle cells, and the human urothelial RT4 cell line. Hypotonic stretch increased ATP release in all four cell types, with porcine urothelial cells exhibiting a 3.8 ± 1.3-fold and RT4 cells a 2.0 ± 0.5-fold increase relative to control levels. Similarly, depletion of extracellular calcium ions (Ca^2+^) stimulated ATP release from porcine urothelial cells and RT4 cells, yielding 5.4 ± 2.9-fold and 2.4 ± 0.8-fold increases, respectively. Blockade of Cx43 channels with a Cx43 mimetic peptide (peptide 5) and Cx45 channels with a Cx45 mimetic peptide reduced ATP release induced by stretch and Ca^2+^ depletion in porcine urothelial cells by 50% and 67%, respectively. These blockers also reduced ATP release in RT4 cells. The contributions of Cx43 and Cx45 to ATP release were less prominent in suburothelial and detrusor muscle cells compared to urothelial cells.

**Conclusion:**

These findings highlighted ATP’s role as an autocrine/paracrine signaling molecule acting on purinergic receptors during bladder distension and suggested that Cx hemichannels regulate ATP release through mechanotransduction and Ca^2+^-sensitive pathways, providing new insights into bladder sensory mechanisms.

## 1. Introduction

Over the past two decades, the role of adenosine triphosphate (ATP) in mediating bladder sensation under both physiological and pathophysiological conditions has become well recognized.^1^ ATP, released from the urothelium in response to bladder wall distension, mediates the sensation of bladder fullness and transmits sensory information to the central nervous system through the activation of purinergic P2X2/3 receptors on sensory afferent neurons.^2^,^3^ Enhanced ATP release has been reported in patients with bladder pathologies associated with urinary urgency and frequency, including interstitial cystitis/bladder pain syndrome,^4^,^5^ and bladder overactivity.^6^,^7^

ATP release in the bladder occurs through both vesicular and non-vesicular ATP mechanisms. Vesicular ATP release is mediated by the activation of vesicular exocytosis,^8^ while non-vesicular mechanisms involve the direct transport of ATP across cell membranes through specific channels and pathways. We and other researchers have identified several non-vesicular ATP release pathways in the bladder across different species, including pannexin-1 channels,^9^,^10^ transient receptor potential vanilloid (TRPV1 and TRPV4) channels,^11^,^12^ acid-sensing ion channels,^13^ the Piezo 1 channel,^14^ epithelial sodium ion channels,^15^ calcium homeostasis modulator (CALHM) 1,^9^ and ATP-binding cassette transporters.^16^

Another potential contributor to ATP release is the connexin (Cx) family of proteins. Cxs are transmembrane proteins that form gap junctions between adjacent cells to regulate cell-to-cell communication. However, some Cxs, including Cx 43 (Cx43) and Cx 45 (Cx45), can also form functional hemichannels that release small molecules, such as ATP, into the extracellular environment.^17^,^18^ These hemichannels remain closed under resting physiological conditions, but stimuli, such as reduced extracellular calcium ion (Ca^2+^) concentration^19^ and mechanical stretch,^20^ are capable of opening their pores. Previous studies have shown that Cx hemichannels, in particular Cx43, mediate ATP release in many cell types.^21^-^23^ Although increased expression of Cx43 and Cx45 has been demonstrated in patients with symptoms of urinary urgency and frequency,^24^ their functional role in bladder ATP release remains to be established.

This study addressed these knowledge gap by: (i) identifying the expression patterns of Cx43 and Cx45 in the porcine bladder, a well-recognized model for studying human bladder function, and (ii) investigating their roles in mediating ATP release in response to hypotonic stretch and extracellular Ca^2+^ depletion using primary cell cultures derived from porcine bladder urothelial, suburothelial, and detrusor muscle cells, as well as the human RT4 urothelial cell line.

## 2. Materials and methods

### 2.1. Cell isolation and culture

Bladders excised from female pigs (6–9 months old, *n* = 60) at a local abattoir were transported to the laboratory on ice. Ethical approval was not required, as the bladder specimens were obtained from pigs that had been processed for commercial meat production. Fat tissue was removed from the bladder surface, and the bladders were rinsed with Krebs-Henseleit (K-H) solution (composition in mM: sodium chloride [NaCl] 118, sodium bicarbonate 25, potassium chloride 4.7, monopotassium phosphate 1.2, calcium chloride 2.5, magnesium sulfate 1.2, and D-glucose 11.7), bubbled with carbogen to stabilize the pH at 7.4. The solution was supplemented with 1% antibiotic-antimycotic solution (containing 10,000 units/mL penicillin, 10,000 μg/mL of streptomycin, and 25 μg/mL of amphotericin B).

The method for bladder cell isolation and culture has been described priorly in detail.^9^,^25^ Briefly, urothelial cells were scraped from the luminal surface of the bladder mucosa and collected into RPMI 1640 culture medium (Sigma Aldrich, Merck KGaA, Germany) supplemented with 10% fetal bovine serum (FBS; Thermo Fisher Scientific, USA) and 1% antibiotic-antimycotic solution (Sigma Aldrich, Merck KGaA, Germany). To remove residual urothelial cells, 0.25% trypsin-ethylenediaminetetraacetic acid (EDTA; Sigma Aldrich, Merck KGaA, Germany) was applied to the luminal surface for 5 min at 37°C, followed by further scraping to facilitate dissociation of urothelial cells from the mucosal layer. The cells were pelleted by centrifugation at 750 × g for 5 min and resuspended in RPMI. Urothelial cells were plated into 48-well plates at approximately 10^6^ cells per well and incubated at 37°C in 5% carbon dioxide (CO_2_) until 70–80% confluence.

The bladder tissue (excluding the urothelium) was rinsed with the K-H solution and dissected into suburothelial and detrusor muscle layers. Under a dissection microscope, loose connective tissue, residual muscle bundles, and blood vessels were removed from the suburothelial layer. The macroscopically “pure” suburothelial and detrusor muscle layers were minced and treated with 0.25% trypsin-EDTA for 30 min at 37°C. Trypsin was neutralized with an equal volume of RPMI culture medium, and cells were filtered through a 100-μm pore size cell strainer. The cells were pelleted at 750 × g for 5 min, resuspended in RPMI, and plated into 48-well plates as described above for urothelial cells.

Human urothelial RT4 cells (91091914, Sigma Aldrich, Merck KGaA, Germany) were cultured in McCoy’s 5A medium supplemented (Thermo Fisher Scientific, USA) with 10% FBS, 1% GlutaMAX (Thermo Fisher Scientific, USA), and 1% antibiotic-antimycotic solution (containing 10,000 U/mL penicillin, 10,000 g/mL streptomycin, and 25 g/mL amphotericin B) at 37°C in 5% CO_2_. Confluent cells were passaged by incubation with 0.25% trypsin-EDTA for 5 min at 37°C and then plated onto 48-well plates for ATP release studies (approximately 3–5 days post-passage).

### 2.2. Immunohistochemistry

Segments of porcine bladder specimens (dome and lateral regions, *n* = 4) were fixed in Zamboni’s solution, embedded in paraffin, sectioned (4 μm), and mounted on poly-L-lysine-coated slides. Paraffin was removed with 100% xylene, and sections were rehydrated with sequential ethanol solutions (100%, 95%, 70%, and 50%) for 2 min each. Slides were washed with phosphate-buffered saline (PBS; University of New South Wales, Australia; 0.1 M, pH 7.4) and incubated with 3% hydrogen peroxide for 5 min to block endogenous peroxidase activity. Non-specific binding sites were blocked by incubating the slides in PBS containing 10% goat and donkey serum for 30 min at room temperature.

**Figure A1:**
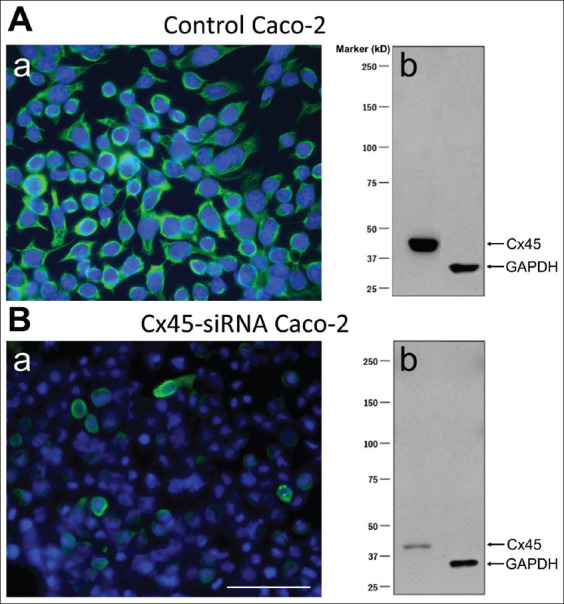
Verification of the specificity of the connexin 45 (Cx45) antibody in human intestinal epithelial Caco-2 cells. (A[a]) shows control Caco-2 cells with dense Cx45 immunofluorescent staining (green), corresponding to a strong Cx45 band in the Western blot (A[b]). In contrast, Caco-2 cells with Cx45 gene knockdown through small interfering RNA (siRNA) exhibit a marked reduction in both Cx45 immunoreactivity (B[a]) and Western blot signal (B[b]). These results confirm the specificity of the Cx45 antibody used in this study. Both image panels are shown at the same magnification (20×), with the scale bar representing 100 μm.

For cellular localization of Cx43 and Cx45, fluorescent immunohistochemistry was conducted in intact porcine bladder sections using primary antibodies against Cx43 (C6219, Sigma-Aldrich, Merck KGaA, Germany; 1:100 dilution) and Cx45 (AB1745, Millipore, Merck KGaA, Germany; 1:100 dilution). The Cx43 antibody used in the present study was previously validated in Cx43 knockout models.^26^ We validated the specificity of the Cx45 antibody (AB1745, Millipore, Merck KGaA, Germany) using the small interfering RNA technique in Caco-2 cells (European Collection of Authenticated Cell Cultures, UK). Following Cx45 gene silencing, Cx45 immunoreactivity (IR) was reduced by 90% in Caco-2 cells, confirming the antibody’s specificity (Figure A1).

Cx43 and Cx45 were also double-labeled with primary marker antibodies: AE1/AE3, a cytokeratin marker (M3515, Dako, Agilent, USA; 1:200 dilution); vimentin, a cytoskeleton filament marker (M072529-2, Dako, Agilent, USA; 1:200 dilution); and α-smooth muscle actin (α-SMA), a contractile protein marker (M085129-2, Dako, Agilent, CA, USA; 1:200 dilution). Slides were incubated overnight with primary antibodies at room temperature, washed (3 × 10 min) in tris-buffered saline (TBS; University of New South Wales, Australia), and incubated with secondary antibodies, green-fluorescent Alexa Fluor^®^ 488 (ab150077, Abcam, Australia; 1:200 dilution) and red-fluorescent Alexa Fluor^®^ 594 (ab150116, Abcam, Australia; 1:200 dilution), for 1 h at room temperature. Slides were washed again with TBS (3 × 10 min) and coverslipped using 4’,6-diamidino-2-phenylindole (DAPI; P36962, Thermo Fisher Scientific, USA) as a nuclear and chromosome counterstain.

For immunocytochemistry, urothelial, suburothelial, and detrusor muscle cells isolated from porcine bladder, as well as human urothelial RT4 cells, were cultured on coverslips for 10–14 days and fixed with a 95% ethanol and 5% acetic acid solution for 10 min at −20°C. The protocols for detecting IR of Cx43, Cx45, AE1/AE3, vimentin, and α-SMA were identical to those used for tissue sections. In each experiment, a negative control was included using the secondary antibody alone; these consistently showed no immunoreactive staining.

Immunoreactive images were visualized and captured using an Olympus BX51 microscope (Olympus Corporation, Japan) with 20× and 40× objectives and Neurolucida software (MBF Bioscience, USA).

## 2.3. ATP release assays

ATP release assay was performed as previously described.^9^,^25^ In brief, confluent urothelial, suburothelial, and detrusor muscle cell cultures (10–14 days), derived from porcine bladder, as well as human urothelial RT4 cells, were gently exposed to 300 μL of K-H solution (with or without inhibitors) for 1 h at 37°C. After incubation, 150 μL of supernatant was collected from each well to measure the basal ATP level.

To assess ATP release in response to hypotonic stretch, cells were treated for 10 min with 150 μL of K-H solution containing ~50% reduced NaCl. ATP release under extracellular Ca^2+^-free conditions was assessed by replacing 100 μL of the supernatant with Ca^2+^-free K-H solution (containing 3.5 mM ethylene glycol-bis [β-aminoethyl ether]-N,N,N’,N’-tetraacetic acid; final extracellular Ca^2+^ concentration ~17 nM, calculated using WEBMAXC: https://somapp.ucdmc.ucdavis.edu/pharmacology/bers/maxchelator/webmaxc/webmaxcS.htm). ATP release was measured 2 min after exposure to the Ca^2+^-free K-H solution (denoted as [Ca^2+^]_0_).

ATP concentrations were measured using the ATP Bioluminescence Assay Kit (MAK133-1KT, Sigma-Aldrich, Merck KGaA, Germany) on a GloMax 20/20 Luminometer (Berthold Technologies, GmbH&Co. KG, Germany). The luminescence reading from each well was converted to ATP concentration against a standard curve. ATP release was calculated as the percentage of ATP released relative to the basal level in the same well before exposure to hypotonic or [Ca^2+^]_0_ solutions.

Peptide 5 (VDCFLSRPTEKT, manufactured by Mimotopes, Australia) is a Cx43 mimetic peptide corresponding to a region of the second extracellular loop of Cx43 and functions as a Cx43 hemichannel blocker. The Cx45 mimetic peptide (referred to as the Cx45 peptide in this study) corresponds to amino acids 202–217 of Cx45 (QVHPFYVCSRLPCPHK, manufactured by China Peptides, China) and acts as a Cx45 channel blocker. The effects of Cx43 and Cx45 channel blockers on ATP release were assessed under basal conditions as well as during hypotonic stretch and [Ca^2+^]_0_ conditions. Under basal conditions, ATP levels remained comparable to control values across all concentrations of Cx43 and Cx45 channel blockers tested.

### 2.4. Statistical analysis

Data were analyzed using GraphPad Prism (version 9, GraphPad Software, Inc., USA). Results are expressed as mean ± SD, with *n* representing the number of animals used, except for RT4 cells, where *n* denotes the number of replicate experiments. One-way analysis of variance (ANOVA) followed by Sidak’s multiple comparisons test was used for comparisons among multiple groups. For data involving Cx43 and Cx45 blockers, where both concentration and treatment condition were considered as factors, a two-way ANOVA followed by Sidak’s multiple comparisons test was performed.

Given the exploratory nature of the study, the calculated *p*-values are interpreted as descriptive indicators of potential differences rather than definitive hypothesis-testing results. All tests were two-tailed, and *p*<0.05 was considered indicative of potential significance.

## 3. Results

### 3.1. Cx43 and Cx45 expression in the porcine bladder

**Figure 1:**
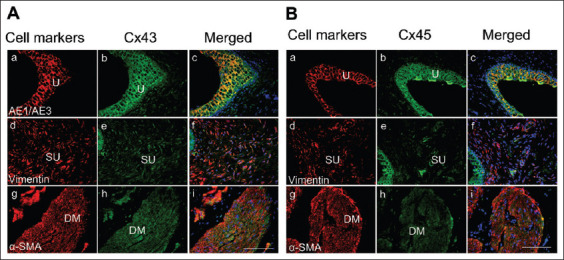
Double labeling of connexin (Cx) 43 and Cx45 antibodies (green) with cell marker antibodies (red) in porcine bladder tissues. Experiments were performed on four animals, and the results were consistent across all specimens. A similar cellular distribution was observed for Cx43 (A) and Cx45 (B) in the porcine bladder. In the urothelium (U) layer, Cx43-immunoreactive (IR) and Cx45-IR signals were expressed throughout all three layers of the urothelium (umbrella, intermediate, and basal cells) and co-localized with AE1/AE3 (a-c). In the lamina propria of the suburothelial (SU) layer, Cx43-IR and Cx45-IR co-localized with vimentin-positive spindle-shaped cells, likely corresponding to suburothelial myofibroblasts (d-f). Positive Cx43-IR and Cx45-IR were also observed in the detrusor muscle (DM), where they co-localized with α-smooth muscle actin (α-SMA) (g-i). Cell markers used: Epithelial cytokeratin marker AE1/AE3, cytoskeleton filament maker vimentin, and smooth muscle marker α-SMA. Blue staining indicates nuclei stained with 4’,6-diamidino-2-phenylindole. All panels are shown at the same magnification (20×). Scale bars represent 100 μm.

Immunofluorescence staining for Cx43 and Cx45 showed their expression in urothelial, suburothelial, and detrusor muscle layers of porcine bladder tissues (Figure 1). Double labeling of Cx43 or Cx45 with cell-specific markers, that is, the epithelial cytokeratin marker AE1/AE3, the cytoskeletal filament vimentin, and the contractile protein α-SMA, was performed to confirm the identities of urothelial, suburothelial, and detrusor muscle cells. Co-staining with AE1/AE3 revealed that both Cx43 and Cx45 were expressed throughout all three layers of the urothelium: umbrella, intermediate, and basal cell layers (Figure 1A[a-c] and 1B[a-c]). In the lamina propria, Cx43-IR and Cx45-IR were co-localized with vimentin-positive spindle-shaped cells that likely correspond to suburothelial myofibroblasts (Figure 1A[d-f] and 1B[d-f]). Moreover, positive Cx43-IR and Cx45-IR were also observed on the surface of detrusor muscle cell membrane, co-localized with α-SMA (Figure 1A[g-i] and 1B[g-i]).

Abbreviation: GAPDH: Glyceraldehyde 3-phosphate dehydrogenase.

**Figure 2:**
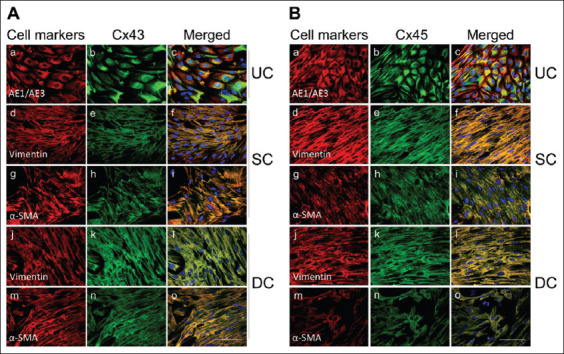
Immunocytochemistry of cultured urothelial, suburothelial, and detrusor muscle cells. Fluorescent immunocytochemical characterization was performed on cultured porcine bladder cells using cell marker antibodies (red) and double labeling with connexin (Cx) 43 (A) and Cx45 (B) antibodies (green). The epithelial cell marker AE1/AE3 was observed exclusively in urothelial cells (a). Vimentin and α-smooth muscle actin (α-SMA) were positive in both suburothelial (d and g) and detrusor muscle (j and m) cell cultures, but were absent in urothelial cells (data not shown). Cx43- immunoreactivity (A) and Cx45-immunoreactivity (B) were detected in cultured urothelial (b), suburothelial (e and h), and detrusor muscle cells (k and n). Cx43 and Cx45-immunoreactivities co-localized with cell marker AE1/AE3 (c), vimentin (f and l), and α-SMA (i and o). Blue staining indicates nuclei labeled with 4’,6-diamidino-2-phenylindole. All panels are shown at the same magnification (20×), with scale bars representing 100 μm. Abbreviations: DC: Detrusor cells; SC: Suburothelial cells; UC: Urothelial cells.

The expression of Cx43 and Cx45 in isolated cultured porcine urothelial, suburothelial, and detrusor muscle cells confirmed the localization of these Cx proteins with individual cell populations (Figure 2). As expected, the urothelial cell marker AE1/AE3-IR was densely expressed on urothelial cells (Figure 2A[a] and 2B[a]), whereas suburothelial and detrusor muscle cells showed no positive AE1/AE3 staining (data not shown). Consistent with expectations, suburothelial and detrusor muscle cells displayed strong IR for both vimentin (Figure 2A[d,j] and 2B[d,j]) and α-SMA (Figure 2 A[g,m] and Figure 2B[g,m]).

In cultured urothelial, suburothelial, and detrusor muscle cells, Cx43 and Cx45 showed a cellular distribution profile similar to that observed in intact porcine bladder segments. In urothelial cells, Cx43-IR (Figure 2A[b,c]) and Cx45-IR (Figure 2B[b,c]) were prominently distributed throughout the cytoplasm and plasma membrane, with a much stronger presence compared to suburothelial myofibroblasts (Figure 2A[e,f,h,i] and 2B[e,f,h,i]) and detrusor muscle cells (Figure 2A[k,l,n,o] and 2B[k,l,n,o]). In addition, the localization of Cx43 and Cx45 appeared to vary with the stage of cell division, as positive signals for both proteins were also observed on the nuclear membrane and within the nucleus in some cells (data not shown).

### 3.2. Involvement of Cx43 and Cx45 channels in ATP release in porcine bladder cells

**Figure 3:**
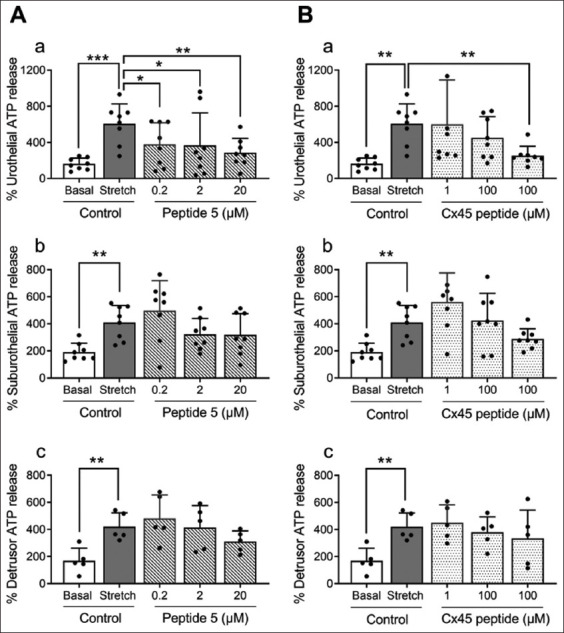
Effects of the connexin (Cx) 43 blocker peptide 5 and the Cx45 blocker Cx45 peptide on stretch-induced adenosine triphosphate (ATP) release in cultured porcine bladder urothelial, suburothelial, and detrusor muscle cells. Hypotonic stretch significantly increased ATP release in all three types compared to their respective basal controls (*n* = 5–8; **p<0.01, ***p<0.001; two-way analysis of variance followed by Sidak’s multiple comparisons test; A[a-c] and B[a-c]). Peptide 5 (A) and Cx45 peptide (B) appeared to partially inhibit the stretch-induced increase in a concentration-dependent manner in cultured urothelial (Aa and Ba), suburothelial (Ab and Bb), and detrusor muscle (A[c] and B[c]) cells. However, statistically significant reductions were observed only in urothelial cells for both peptide 5 and Cx45 peptide (*p<0.05, **p<0.01, respectively; A[a] and B[a]). Results are expressed as mean ± SD.

Stretch induced by a hypotonic solution increased ATP release from cultured porcine bladder urothelial cells to 3.8 ± 1.3-fold of the control level (Figure 3A[a] and 3B[a]), consistent with previous studies.^9^,^25^ The Cx43 channel blocker, peptide 5 (at 0.2, 2, and 20 μM), reduced stretch-induced ATP release from urothelial cells in a concentration-dependent manner, with a maximum inhibition of 53.1% at 20 μM (*n* = 8–10; Figure 3A[a]). Similarly, the Cx45 channel blocker, referred to as Cx45 peptide (at 1, 10, and 100 μM), inhibited stretch-induced ATP release from urothelial cells by 58.4% at 100 μM (*n* = 8–10; Figure 3B[a]).

A similar trend was observed in porcine suburothelial cells, where stretch-induced ATP release was partially reduced by peptide 5 (22% reduction at 20 μM; Figure 3A[b]) and Cx45 peptide (29.9% reduction at 100 μM; Figure 3B[b]), although these reductions were not statistically significant. Likewise, in porcine detrusor muscle cell cultures, peptide 5 (26% reduction at 20 μM; Figure 3A[c]) and Cx45 peptide (20.5% reduction at 100 μM; Figure 3B[c]) exhibited a similar trend without reaching statistical significance.

**Figure 4:**
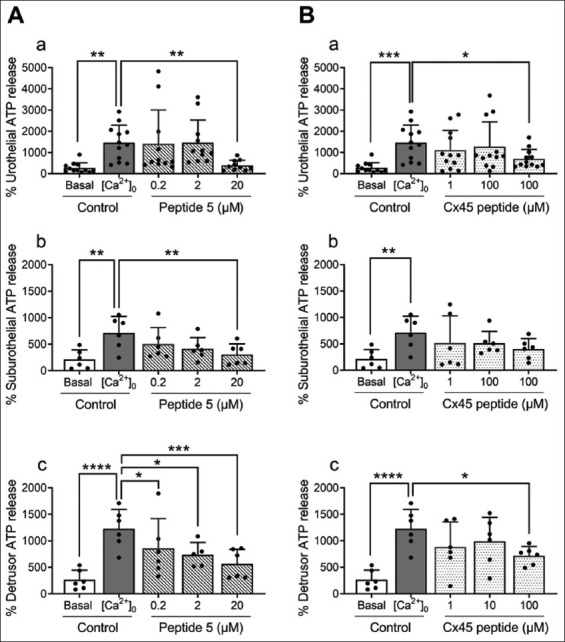
Effects of the connexin (Cx) 43 blocker peptide 5 and the Cx45 blocker Cx45 peptide on adenosine triphosphate (ATP) release under extracellular calcium ion (Ca^2+^)-depleted conditions in cultured porcine bladder urothelial, suburothelial, and detrusor muscle cells. Extracellular Ca^2+^-depleted conditions ([Ca^2+^]_0_, ~17 nM) increased ATP release from all three cell types compared to their respective basal controls (*n* = 6–12; **p<0.01, ***p<0.001, ****p<0.0001; two-way analysis of variance followed by Sidak’s multiple comparisons test; (Aa-c and Ba-c). Peptide 5 (A) virtually completely abolished [Ca^2+^]_0_-evoked ATP release in urothelial (A[a]), suburothelial (A[b]), and detrusor muscle (A[c]) cells (*p<0.05, **p<0.01, ***p<0.001). Cx45 peptide (B) partially inhibited [Ca^2+^]_0_-evoked ATP release in cultured urothelial (B[a]), suburothelial (B[b]), and detrusor muscle (B[c]) cells. However, a statistically significant reduction was only observed in urothelial cells at the highest concentration of Cx45 peptide (100 μM; *p<0.05; B[a]). Results are expressed as mean ± standard deviation.

Depletion of extracellular Ca^2+^ increased ATP release from porcine bladder urothelial cells to 5.4 ± 2.9-fold relative to control levels (Figure 4A[a] and 4B[a]). The application of peptide 5 reduced [Ca^2+^]_0_-induced ATP release by approximately 54% at 20 μM (*n* = 6–10; Figure 4A[a]). Peptide 5 also inhibited ([Ca^2+^]_0_)-induced ATP release from suburothelial (Figure 4A[b]) and detrusor muscle cells (Figure 4A[c]) in a concentration-dependent fashion.

Similarly, Cx45 peptide (100 μM) reduced [Ca^2+^]_0_-evoked ATP release from urothelial cells by 66.9% (Figure 4B[a]). In suburothelial cells (Figure 4B[b]) and detrusor muscle cells (Figure 4B[c]), Cx45 peptide revealed a trend toward inhibition of ATP release, although the effect was less pronounced.

### 3.3. Expression of Cx43 and Cx45 and ATP release in human RT4 cells

**Figure 5:**
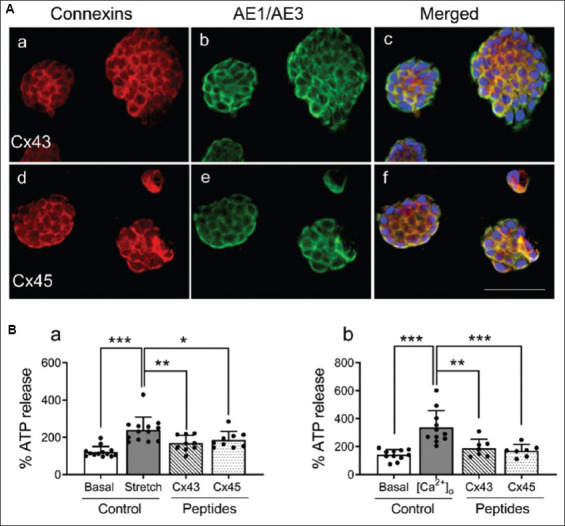
Connexin (Cx) 43 and Cx45 expression in human urothelial RT4 cells and their involvement in stretch-induced and extracellular calcium ion (Ca^2+^)-depletion-induced adenosine triphosphate (ATP) release. (A). Double labeling of Cx43 and Cx45 antibodies (red) with the cytokeratin marker AE1/AE3 antibody (green) in RT4 cells revealed that Cx43-IR (A[a]) and Cx45-IR (A[d]) are primarily localized to the cell membranes. AE1/AE3-positive staining (A[b,c] and A[e,f]) confirms the epithelial nature of the urothelial RT4 cells. Blue staining represents nuclei stained with 4’,6-diamidino-2-phenylindole. All panels are shown at the same magnification (20×), with the scale bar representing 100 μm. (B) Both hypotonic stretch (B[a]) and extracellular Ca^2+^-depletion (B[b]) increased ATP release from RT4 cells compared to basal controls (*n* = 9–13 replicates; ***p<0.001; one-way analysis of variance followed by Sidak’s multiple comparisons test). In the presence of the Cx43 channel blocker peptide 5 (20 μM) and the Cx45 channel blocker (Cx45 peptide; 100 μM), both stretch-induced ATP release (B[a]) and [Ca^2+^]_0_-induced ATP release (B[b]) were significantly inhibited (*n* = 7–9 replicates; *p<0.05, **p<0.01, ***p<0.001).

Intense Cx43-IR (Figure 5A[a]) and Cx45-IR (Figure 5A[d]) were observed in human urothelial RT4 cells, with expression patterns similar to those seen in porcine urothelial cells. The immunoreactive staining was dense within the cytoplasm and along the cell membranes. RT4 cells also displayed strong IR for the epithelial cell marker AE1/AE3, confirming their epithelial identity (Figure 5A[b,e]). This marker was co-expressed with Cx43 (Figure 5A[c]) and Cx45 (Figure 5A[f]).

In RT4 cells, stretch resulted in an approximately 100% increase in ATP release compared to control. As shown in Figure 5B[a], this stretch-induced ATP release was attenuated by 30% with peptide 5 (20 μM) and by 22.5% with Cx45 peptide (100 μM). Under extracellular Ca^2+^-free ([Ca^2+^]_0_) conditions, ATP release increased by approximately 140% relative to control and was inhibited by 44% with peptide 5 and by 50% with Cx45 peptide (Figure 5B[b]).

## 4. Discussion

The present study demonstrated the expression of Cx43 and Cx45 in the porcine bladder and provides substantial evidence for their role in mediating ATP release from urothelial cells. Until now, most studies investigating Cx43 and Cx45 in the urinary bladder have focused on their expression in bladder pathologies^24^,^27^,^28^ and their involvement in gap junction formation.^28^-^30^ In this study, the porcine bladder, an appropriate model for studying human bladder function,^31^ and human urothelial RT4 cells were used to confirm the functional role of Cx43 and Cx45 in mediating ATP release in the urinary bladder.

Cx43 and Cx45 staining was observed in all three layers of the porcine bladder, with particularly dense staining on the urothelium. A similar dense staining pattern was also evident in human urothelial RT4 cells. The cellular expression of Cx43 and Cx45 was localized to the cytoplasm and cell membranes of urothelial cells, with occasional localization to the nuclear membrane or within the nuclei. These findings suggest that Cx43 and Cx45 function as active channels in the bladder, with their localization potentially varying depending on different stimuli and stages of the cell cycle.

Double labeling of Cx43 and Cx45 with the cytokeratin marker AE1/AE3 revealed that these Cx proteins are expressed throughout all three layers of the urothelium (umbrella, intermediate, and basal layers). This finding suggests an important role for Cx43 and Cx45 in the urothelium, potentially facilitating the release of signaling molecules to both the lamina propria and the bladder lumen.

In the suburothelial space, Cx43 and Cx45 IR co-localized with vimentin-positive spindle-shaped cells, which are likely suburothelial myofibroblasts.^25^ In the lamina propria, these myofibroblasts are located in close proximity to suburothelial afferent nerve terminals, and ATP released from the basolateral urothelial surface or suburothelial myofibroblasts may influence sensory afferent nerve activity.^32^ Furthermore, given that Cx43 and Cx45 are gap junction proteins,^17^ they are likely involved in regulating cell-to-cell communication, possibly providing a mechanism for coordinating signals from the urothelium to the detrusor muscle.^32^

Numerous studies have demonstrated that the urothelium releases ATP in response to stretch of the bladder wall.^2^,^9^,^25^ In the present study, hypotonic medium, a well-established model for mimicking mechanical stretch,^9^,^25^ was used to trigger stretch-induced ATP release from cultured cells. Peptide 5 and the Cx45 mimetic peptide reduced ATP release from urothelial cells in response to hypotonic stretch, suggesting that Cx43 and Cx45 not only play important roles in regulating cell-to-cell communication in the urinary bladder^28^,^32^ but also form functional hemichannels that may have a physiologically relevant role in bladder ATP release.

This study also demonstrated that depleted extracellular Ca^2+^ triggers ATP release, consistent with previous findings in rabbit urinary bladder strips^2^ and isolated urothelial, suburothelial, and detrusor muscle cell cultures from the porcine bladder.^9^ Cx hemichannels normally remain closed under physiological extracellular Ca^2+^ levels (approximately 1.1–1.3 mM). However, exposure to extracellular solutions with reduced or zero Ca^2+^ activates these channels, facilitating ATP release through this pathway.^33^

In all four cell types, porcine urothelial, suburothelial, and detrusor muscle cells, and human urothelial RT4 cells, depleted extracellular Ca^2+^ triggered ATP release, which was blocked by peptide 5. This finding highlights the essential role of Cx43 hemichannels in mediating ATP release. However, the Cx45 mimetic peptide only inhibited ATP release from cultured urothelial cells (both porcine and RT4) in response to extracellular Ca^2+^ depletion. This suggests that Cx45 hemichannels may play a critical functional role in the urothelium, but not in the suburothelial or detrusor muscle layers.

It is noteworthy that both histological and functional data support an essential role for Cx43 and Cx45 in urothelium. The intense expression of C43 and Cx45 in all three cell types of urothelium (umbrella, intermediate, and basal cells) indicates that these channels may facilitate the release of ATP and other small molecules to both the lamina propria and the bladder lumen. This extends the previous notion that ATP is released from the urothelium through both apical^8^,^10^ and basolateral routes.^3^ Apical ATP release can activate purinergic P2X/P2Y receptors on the luminal cell membrane, triggering ATP-mediated ATP release.^8^,^34^ In addition, ATP released from the basal surface of the urothelium can directly activate purinergic receptors on afferent nerve terminals and myofibroblasts in the mucosal area.^3^,^35^

In the present study, peptide 5 and the Cx45 mimetic peptide were able to block ATP release from the urothelium but were less effective in suburothelial and detrusor muscle cells, indicating that these Cxs may perform different functions in these cell types. The ability of Cxs to form gap junctions is well established, with several studies showing that lamina propria myofibroblasts^32^ and detrusor muscle cells^36^ are electrically coupled through gap junctions. This coupling appears to play a major role in mediating intrinsic contractile activity in the bladder.^32^ Alterations in Cx43 and Cx45 expression in the detrusor layer have also been reported in patients experiencing symptoms of urinary urgency and frequency, as well as in rat models of overactive bladder.^24^,^28^

## 5. Conclusion

To the best of our knowledge, this is the first study to investigate the functional role of Cx43 and Cx45 in mediating ATP release from porcine bladder urothelial, suburothelial, and detrusor muscle cells, as well as from human urothelial RT4 cells. Based on our findings, we conclude that Cx43 and Cx45, along with several other channels such as pannexin-1 and CALHM1, play a role in stimulating ATP release from the urothelium. Further research is needed to determine whether Cx43 and Cx45 contribute to augmented ATP release in the urine of patients with bladder pathologies such as interstitial cystitis/bladder pain syndrome or overactive bladder. If such a role is confirmed, it could reveal new therapeutic opportunities, as clinical trials have already shown that the application of a Cx43 mimetic peptide gel is effective in treating inflammatory conditions.^37^ Thus, Cx43 and Cx45 mimetic peptides may hold therapeutic potential for the treatment of inflammatory bladder diseases.

## Data Availability

Data obtained from this study are available from the corresponding author on reasonable request.
